# Thermal Contaminants in Coffee Induced by Roasting: A Review

**DOI:** 10.3390/ijerph20085586

**Published:** 2023-04-20

**Authors:** David Silva da Costa, Tânia Gonçalves Albuquerque, Helena Soares Costa, Adriana Pavesi Arisseto Bragotto

**Affiliations:** 1 Faculdade de Engenharia de Alimentos, Universidade Estadual de Campinas, Cidade Universitária, R. Monteiro Lobato 80, Campinas 13083-862, Brazil; 2 Departamento de Alimentação e Nutrição, Instituto Nacional de Saúde Doutor Ricardo Jorge, I.P. Av. Padre Cruz, 1649-016 Lisboa, Portugal; 3 REQUIMTE-LAQV, Faculdade de Farmácia da Universidade do Porto, R. Jorge de Viterbo Ferreira 228, 4050-313 Porto, Portugal

**Keywords:** food processing, Maillard reaction, public health, toxicity, carcinogens, *Rubiaceae*

## Abstract

Roasting is responsible for imparting the main characteristics to coffee, but the high temperatures used in the process can lead to the formation of several potentially toxic substances. Among them, polycyclic aromatic hydrocarbons, acrylamide, furan and its derivative compounds, α-dicarbonyls and advanced glycation end products, 4-methylimidazole, and chloropropanols stand out. The objective of this review is to present a current and comprehensive overview of the chemical contaminants formed during coffee roasting, including a discussion of mitigation strategies reported in the literature to decrease the concentration of these toxicants. Although the formation of the contaminants occurs during the roasting step, knowledge of the coffee production chain as a whole is important to understand the main variables that will impact their concentrations in the different coffee products. The precursors and routes of formation are generally different for each contaminant, and the formed concentrations can be quite high for some substances. In addition, the study highlights several mitigation strategies related to decreasing the concentration of precursors, modifying process conditions and eliminating/degrading the formed contaminant. Many of these strategies show promising results, but there are still challenges to be overcome, since little information is available about advantages and disadvantages in relation to aspects such as costs, potential for application on an industrial scale and impacts on sensory properties.

## 1. Introduction

The coffee production chain has a significant influence on the world economic sector, making it the second most marketable commodity in the world [1,2]. According to data from the International Coffee Organization (ICO), about 129 million bags of 60 kg of beans were exported worldwide in 2021–2022. Brazil, Vietnam, and Colombia are the largest producers and exporters of coffee in the world, with 39, 26, and 12 million bags exported, respectively, in the period of 2021–2022, which is equivalent to 60% of the world’s production [3,4].

Coffee belongs to the botanical family *Rubiaceae*, which has about 500 genera and more than 6000 species [5]. The genus *Coffea* has just over 100 species, and only two members are widely marketed, *Coffea arabica* (about 70%) and *Coffea canephora* (about 30%), with a restricted supply of the species *Coffea liberica* and *Coffea excelsa* (less than 1%) [1,5]. Green coffee has an astringent taste due to the high levels of chlorogenic acids and, after maturation, it is called coffee cherry due to the red phenotype [6,7].

**Figure 1:**
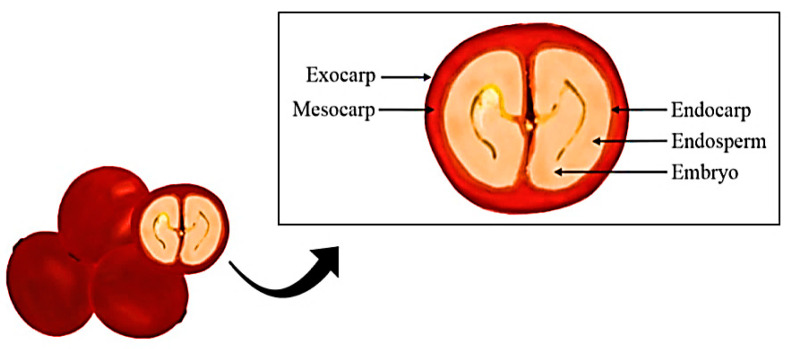
Morphology of the coffee fruit (authorial).

Morphologically, Figure 1 shows that coffee cherry is formed from the exocarp, mesocarp, endocarp, silver skin, and the bean itself, called endosperm. The exocarp, also known as outer skin, is the outermost component of the fruit, formed by a single parenchyma and cellular chloroplast [6,7]. The mesocarp is the pasty intermediate layer of the bean, called the mucilage, which is composed of carbohydrates (pectin, glucose, and fructose), proteins, minerals, polyphenols, and caffeine [8]. The seed (endosperm) has a protective layer known as the spermoderm, called the silver skin. The endosperm has the biological function of protecting the coffee embryo [6,7,8,9].

Coffee is valued for its sensory characteristics, such as a peculiar flavor, but, moreover, for its health benefits. Total polyphenols are bioactive substances found in higher concentrations in the green beans, making coffee a potential food for the prevention of diseases, such as cardiovascular diseases, cancer, and Alzheimer’s disease. Among these bioactive substances, chlorogenic acids, trigonelline, and diterpenes (cafestol) stand out [10,11,12,13,14].

The coffee fruit goes through different stages before it reaches the consumer’s cup. Cultivation, harvesting, processing, and storage are known as preprocessing, and are important steps for standardization and quality [15]. The formation of chemical components responsible for sensory perception, such as flavor, aroma, and color, occurs in roasting, which is a process divided into drying, roasting, and cooling [16]. Roasting is the stage responsible for chemical transformations such as pyrolysis, Maillard reaction, and caramelization, forming flavors, aromas, and pigments characteristic of the product [17]. In roasted coffee, the number of identified volatile chemicals can reach 1000 and, of these, about 300 are heterocyclic organic compounds, which include thiazoles, imidazoles, furans, and pyrazines [18].

Despite the sensory importance of roasting for coffee, several toxic substances can be formed at this stage [1,19,20,21,22,23,24]. Among them, polycyclic aromatic hydrocarbons, acrylamide, furan, furfuryl alcohol, 5-hydroxymethylfufural, α-dicarbonyls and advanced glycation end products, 4-methylimidazole and chloropropanols can be highlighted, which are substances with neurotoxic, carcinogenic, and genotoxic potential, among others. These compounds can be formed from precursors that are naturally present in green coffee (e.g., sugars, amino acids, fatty acids, among others), which undergo chemical transformations at high temperatures. The precursors and routes of formation are different for each contaminant, with some exceptions, and the formed concentrations can be quite high for some substances. The quantification of these compounds normally depends on chromatographic techniques (liquid or gas) coupled to mass spectrometry or other types of detectors. Regulations exist for only a few substances and in a few countries, but as they can pose a risk to human health, the development of mitigation strategies along the coffee production chain is highly desirable [1,20,21,22,23,24,25].

This review aims to present a current and comprehensive overview of the chemical contaminants formed during coffee roasting, including a discussion about mitigation strategies available to decrease the content of these toxicants.

## 2. Materials and Methods

The publication searches were carried out in six bibliographic databases—PubMed, Google Scholar, Web of Science, PubMed10, SciFinder, and Scopus. After the search on each database, duplicate references were deleted. A scan of all possible information regarding contaminants and coffee was performed, leading to articles published from 1985 to 2022. A total of 182 articles were selected based on the titles and abstracts, and their being written in English, Portuguese, or Spanish.

## 3. Coffee

### 3.1. Species

**Figure 2:**
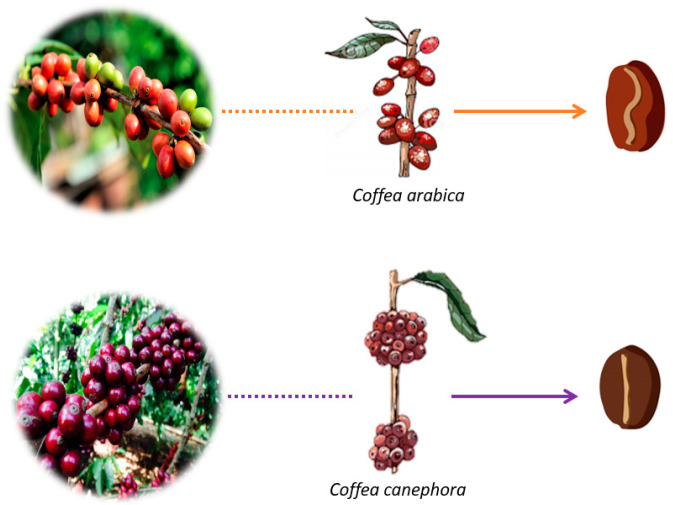
The two most traded coffee species in the world (authorial).

Botanically, coffee belongs to the *Rubiaceae* family, and among the various genera, of which there are about 500, the genus *Coffea* has more than 103 species described in the literature. Only four species are widespread in world trade: *Coffea arabica, Coffea canephora, Coffea liberica,* and *Coffea excelsa*, the first two of which are significantly more expressive [1,5]. Figure 2 shows the morphological differences between the two most highly produced species.

#### 3.1.1. Coffea Arabica

*Coffea arabica* (Arabica coffee), which is native to Ethiopia, is considered the most commercialized coffee species in the world. *Coffea arabica* has superior sensory characteristics, with a sweet taste and strong aroma and with large-scale production worldwide, being consumed pure or in blends (with other species) [1]. This species has a genetic peculiarity when compared with other species, since it is the only tetraploid (the others are diploids) [5,7]. This means that *Coffea arabica* has a 4n configuration, that is, each chromosome has three replicas, thus four basic sets of chromosomes of the genus (*n* = 11), totaling 44 chromosomes [5,25,26].

The species *Coffea arabica* differs from the other species by undergoing fruit maturation in shorter time, ranging from 7 to 9 months; also, Arabica coffee is more susceptible to pests and abiotic variations (temperature, humidity, pH, and altitude) due to its shape [27,28]. Regarding cultivation, this species needs altitudes greater than 600 m and a mild climate (15–22 °C), since without these abiotic factors, the development of the plant is impaired [29]. Additionally, *Coffea arabica* has lower levels of caffeine and soluble solids when compared with *Coffea canephora*, besides its resulting beverage being generally more aromatic and acidic [29].

#### 3.1.2. Coffea Canephora

*Coffea canephora*, popularly known for its variety *Coffea robusta* (Robusta coffee), is native to Central Africa, and its cultivation is popular in Asian countries and in Brazil [30,31]. In contrast to Arabica coffee, Robusta contains higher levels of caffeine, giving it a stronger bitter taste. It is usually used in blends, in which it is combined with Arabica coffee, and may make up to 30% of the final product [32,33]. Robusta coffee gives a higher yield after the roasting process, besides being an essential component of soluble coffees, since it has a higher percentage of soluble solids than Arabic coffee [34].

Since they are diploid, *Coffea robusta* plants have two copies of the basic chromosome number (*n* = 11), resulting in a total of 22 chromosomes per cell nucleus [27]. It presents characteristics of allogamous reproduction and gametophytic self-incompatibility, making cross-fertilization the only form of sexual reproduction of the species [28,35]. Coffee trees of *Coffea robusta* are multi-stem, with a phenological cycle (9–11 months) longer than *Coffea arabica* [31].

Morphologically, its roots are generally more developed than those of Arabica coffee and can absorb even more nutrients and water from the soil, making the plant more resistant to water and nutritional deficiencies [35]. The leaves are larger than the species *Coffea arabica*, with less intense green coloration. The fruits are rounded, red (intense to light), and may be yellow (rare). *Coffea canephora* beans have a shorter shelf life than *Coffea arabica*, and are therefore recalcitrant [35]. *Coffea canephora* is cultivated at lower altitudes (0–900 m), equatorial climates and temperatures ranging from 17 °C (minimum) to a maximum of 31 °C. The species has high levels of caffeine and is less aromatic [36,37,38].

### 3.2. Chemical Composition of the Species Coffea Arabica and Coffea Robusta

Raw, unprocessed, and processed coffee has high chemical complexity [8]. These include the intrinsic components of the beans, which are precursors of the substances responsible for the sensory properties (color, aroma, and flavor) of the coffee beverage, such as proteins (11 to 15%, dry basis), free amino acids (0 to 2%, dry basis), polysaccharides (24 to 55%, dry basis), lipids (9 to 20%, dry basis), trigonelline (0.5 to 1.2%, dry basis), chlorogenic acids (1.2 to 10%, dry basis), and caffeine (0.9 to 2.4%, dry basis) [38]. The most abundant amino acids in raw beans are: alanine (410 to 1400 μg/g), asparagine (280 to 960 μg/g), and phenylalanine (180 to 780 μg/g) [39].

### 3.3. Coffee Preprocessing

Coffee is native to tropical Africa, where it is believed to have evolved as an understory tree [40,41]. The first coffee plantations in the world were shaded, but the plants can also be cultivated in full sun, which leads to the faster maturation of the beans [40,41,42,43]. The cultivation of *Coffea arabica* is generally undertaken in the shade due to its greater susceptibility to temperature variation, whereas *Coffea robusta* can be grown in an environment with higher temperatures, being more suitable for full-sun cultivation [42,43].

Harvesting is a fundamental step in coffee preprocessing, and is associated with the quality of the final product, which is determined by the degree of fruit maturation [44,45]. The presence of immature fruits results in flawed and poor-quality beans, whereas ripe fruits result in a high-quality beverage. This step can be performed manually or mechanically. Depending on the harvesting method, the level of bean defects can increase [44,45,46]. Avoiding the contamination of these fruits by microorganisms, mainly fungi (*Aspergillus, Penicillium,* and *Fusarium*), is also essential, since it can affect both the sensory quality of coffee and the health of the consumer in the case of the presence of mycotoxins [47,48,49].

After harvest, the coffee fruits must undergo a preliminary processing to separate the seeds from the rest of the fruit, consisting of washing and separation processes [50]. They are then separated based on the number and categories of defects, size, and other factors, to assist in the classification of coffee batches. The most used methods are dry and wet processing. The two methods show some variations depending on where the procedure will be performed, but they are, generally, the best known and most widely used [51,52,53,54,55].

### 3.4. Roasting

The green beans, also known as raw coffee, have high rigidity and do not have essential elements appreciated during the tasting of the beverage. With roasting, heat and mass transfer occurs, and the bean becomes more fragile, with darker coloration, allowing the grinding and preparation of the beverage. Coffee beans are roasted at temperatures ranging from 180 to 240 °C [56], and process times can vary from 5 to 15 min. The time–temperature ratio is established according to the desired roasting degree [57,58].

Several types of coffee roasting technology are available today. In terms of roasting techniques, coffee beans are traditionally roasted in batch or continuous systems. Heat can be transferred to the beans by direct contact with hot metal surfaces, runoff (hot air), and radiation. Drum roasters are the most common type of roasters available for domestic and industrial use. They comprise a horizontal rotating cylinder that roasts the coffee beans positioned inside, continuously circulating them, and heating them with hot air pumped through the center of the cylinder or the perforated sides to ensure a uniform roast. Recently, fluid-bed roasting technology has been used in industries, where high-speed hot gas is directed to coffee beans, commonly from the bottom of the roasting machine, so that gases heat and move the beans evenly [59,60].

**Figure 3:**
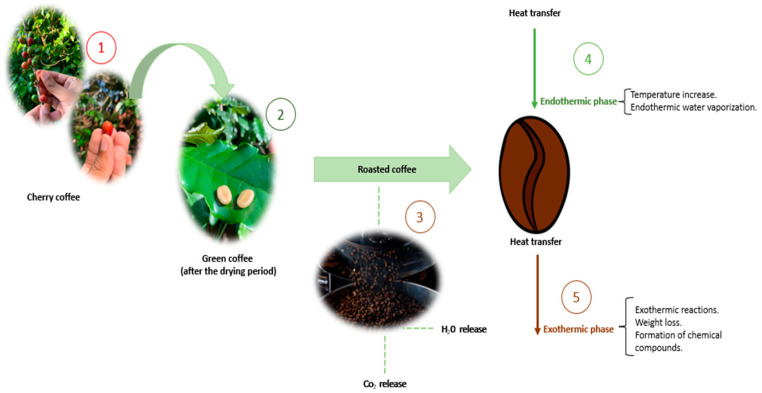
Coffee roasting (authorial). 1: cultivation by hand; 2: drying; 3: roasting; 4: endothermic phase of the roasting; 5: exothermic phase of the roasting.

The roasting is divided into: (i) drying, characterized by the elimination of water (partial) and volatile losses; (ii) roasting, comprising pyrolysis, Maillard reaction, and caramelization, with CO_2_ release; and (iii) cooling, which can occur via cold air or water sprinkling, as shown in Figure 3 [59,60]. Roasting is considered the most important step, contributing to the color, aroma, and flavor of the beans [61,62].

The coffee roasting process comprises three important chemical transformations: pyrolysis, Maillard reaction, and caramelization. Before the roasting process, the bean goes through a drying step until its moisture reaches 8–12%. At the beginning of roasting, the beans start to exhibit a yellowish color, due to the pyrolysis reaction, with moisture and mass loss. Sucrose begins to be transformed into glucose and fructose, accompanied by the degradation of carbohydrates and amino acids. Then, the Maillard reaction occurs, which leads to the formation of several new molecules responsible for the development of the color and aromatic qualities, commonly at temperatures close to 160 °C [63,64].

The Maillard reaction is a compilation of non-enzymatic reactions between reducing sugars and compounds with a free amino group, and occurs mainly in foods submitted to thermal processes [65]. In the first phase of the reaction, the condensation between an amino group from proteins, peptides, or amino acids and the carbonyl group of a reducing sugar occurs [66]. The condensation releases water and forms an unstable Schiff base. In the second stage, the Schiff base rearranges itself, forming Amadori or Heyns products that, in the third and last stage of the reaction, are fractioned and suffer reactions of dehydration, retro-aldolization, and enolization, generating dicarbonyl compounds and unsaturated carbonyl products (heterocyclic, reductone compounds). From this reaction cascade, 5-hydroxomethylfurfural (HMF) and cyclic compounds with high reactivity can be formed. Intermediate compounds such as HMF can be polymerized, forming stable, darkly colored compounds called melanoidins [23].

Finally, in the caramelization stage, residual moisture from the beans evaporates and the first cracking occurs, due to internal pressure (accumulation of formed gases) and expansion of the bean, commonly at temperatures between 170 °C and 200 °C (approximately). At this stage, the sugars and essential oils begin their release on the outside of the bean, leading it to turn a brown hue. The second crack indicates that the roasting process is almost finished, and its completion is dependent on the desired roasting degree. After that, the bean cools down and rests (18–24 h), which is an important step for the release of CO_2_ [63].

### 3.5. Coffee Products

Regarding the degree of roasting, coffees are usually classified into light, medium, and dark roast. Light roast coffee is less bitter, has accentuated acidity, and a softer aroma and flavor when compared with other roasting degrees, and is ideal for preparing espresso coffee. Medium roast coffee has more pronounced acidity than light roast, and is more full-bodied and ideal for filtered coffee (cloth or paper). Dark roast coffee has the lowest sensory gains due to its lower acidity, and it has a light body, is more bitter, and results in a darker beverage [67,68].

Regarding grinding degree, the most common types are fine, medium, and coarse grind coffees. Fine grind is proper for preparing filtered coffee. On the other hand, medium grind can be used for espresso coffee, since the preparation of this beverage occurs under pressure, requiring a higher granulometry to reach adequate concentrations of the beverage. Coarse grinding is more used in the preparation of beverages by percolating or pressing [69,70,71].

Other types of coffee include soluble, flavored, gourmet, organic, and decaffeinated. Soluble coffee production involves the same stages as traditional coffee, such as processing, roasting, and grinding. The differences arise due to the extraction (hydration) and drying (dehydration) processes. Extraction is the hydration stage of the ground powder by immersion in water, aiming to obtain a concentrated beverage while preserving the organoleptic properties. In drying, the extract can be subjected to two processes: spraying (sprinkler) or freezing (freeze drying). This type of coffee has a longer shelf life when compared with traditional coffee, but lower caffeine concentration (about 20% less) [72,73,74,75].

Aromatized coffee is the result of new consumption practices in the world. This type of coffee is obtained from inserting essences into the beans, to add more flavor to the beverage. The aromatization of coffee can occur after the roasting process (cooling), using water vapors with the most diverse types of vaporized essences in the beans, which absorb these droplets since they are still hot [76,77,78,79]. Gourmet coffee has superior quality, which comes from the cultivation and the preparation of the beverage. This type of coffee goes through careful processes before packaging, during which the chosen beans must be ripe and of good quality. Further, it is produced only from the Arabica species [76,77,78,79], which has about 50% less caffeine than Robusta and is much more aromatic [76,77]. Gourmet coffee beans are roasted under milder temperature conditions, since the dark roast is commonly used to disguise defects that are not found in selected beans [80,81].

Organic coffee is produced in crops that do not use pesticides and high-solubility chemical fertilizers (potassium chloride and nitrate), which are replaced by plant and animal organic matter [82,83,84,85]. Decaffeinated coffee contains a maximum level of 3% caffeine. Caffeine extraction occurs before bean roasting, and the methods most commonly used in industries involve organic solvents (ethyl acetate or dichloromethane and water), supercritical fluid (CO_2_), or hot water [86,87,88,89,90,91].

### 3.6. Types of Beverage

**Figure 4:**
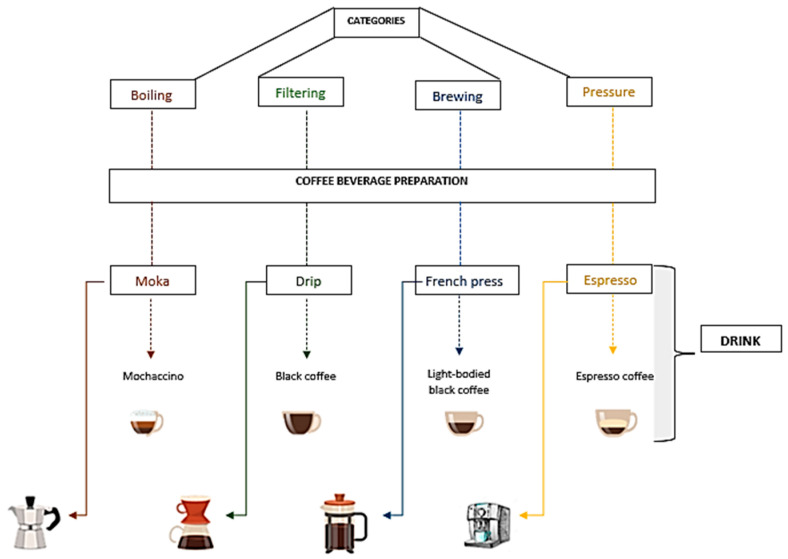
Preparation of coffee beverages (authorial).

The coffee beverage can be prepared in several ways, which can be divided into four main categories, namely: boiling, filtering, infusion, and pressure (Figure 4) [92]. Boiling coffee is most consumed in Europe. It is based on adding the coffee powder into a piece of equipment called a Moka pot, which is heated to bring the water from the bottom of the equipment to boiling and press the liquid coffee into some container, obtaining only the beverage. Filtration is a common method in Brazil, using homemade filters and electric coffee makers, consisting of the addition of hot water, but not boiling, over the coffee powder that is packed in a paper or cloth filter. In the infusion process used in French presses, the coffee is mixed with hot water, but not boiling, and introduced later into a filter to separate the coffee powder from the coffee beverage. In the pressure process, commonly used in the preparation of espresso coffee, the powder is directed to a filter with water pressure at a temperature close to 90 °C for approximately 30 s, producing an aromatic and creamy beverage [92,93,94,95,96,97,98].

## 4. Contaminants Formed in Coffee

The roasting process is the most significant step for commercializing coffee, since it determines the aroma, color, and flavor. However, in addition to the benefits that can be derived from roasting, toxic compounds may be formed due to precursors found in the raw material (proteins, peptides, amino acids, carbohydrates, and reducing sugars). Since coffee is widely consumed worldwide, the formation of contaminants during roasting has become a concern, and may pose a long-term risk to the consumer. Understanding the precursors of these contaminants and their formation and mitigation mechanisms helps in reducing their levels [1,19,20,21,22,23,24].

**Figure 5:**
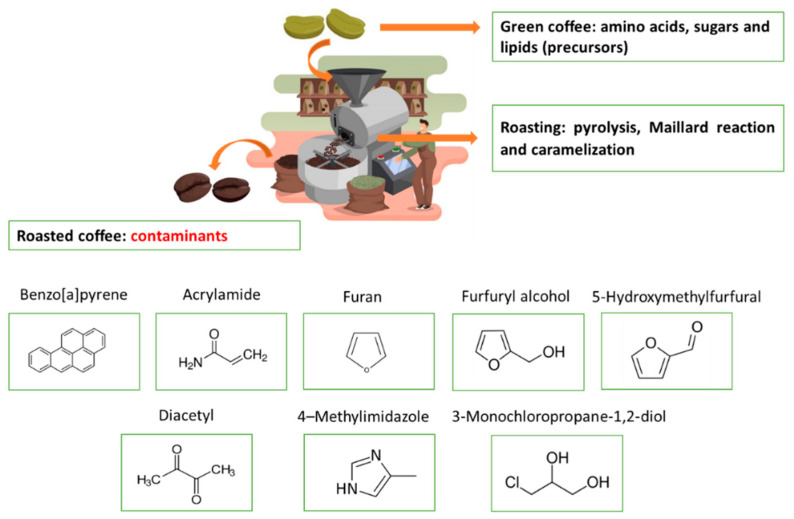
Thermal contaminants in coffee induced by roasting (authorial).

Compounds such as polycyclic aromatic hydrocarbons, acrylamide, furan and derivatives, α-dicarbonyls and advanced glycation end products, 4-methylimidazole and chloropropanols, which are associated with different adverse effects such as neurotoxicity, carcinogenicity, and genotoxicity, are examples of the contaminants formed during coffee roasting (Figure 5) [1,19,20,21,22,23,24].

### 4.1. Polycyclic Aromatic Hydrocarbons

Polycyclic aromatic hydrocarbons (PAHs) are a group of more than 200 hydrophobic compounds consisting of two or more aromatic rings containing carbon and hydrogen atoms, formed from pyrolysis and the incomplete combustion of organic matter [99,100]. In food, the formation of PAHs occurs during high-temperature processes, such as barbecuing, smoking, and roasting. These compounds have lipophilic characteristics due to their aromatic polynuclear components and structures with conjugated double bonds [100].

Molecularly, PAHs can be divided into two groups: (i) low molecular weight, with structures containing up to three fused aromatic rings, and (ii) high molecular weight, with structures containing four or more aromatic rings [101]. Among the PAHs formed in coffee processing, benzo(a)pyrene (Figure 5) stands out as one of the most studied compounds, due to its classification as a carcinogen to humans (group 1) by the International Agency for Research on Cancer (IARC) [99,100,101,102,103,104].

PAHs form from organic precursors, such as amino acids, lipids and carbohydrates, during heat treatment, such as roasting. However, elucidating the exact mechanisms that lead to PAH synthesis in roasted coffee is still a challenge due to the complexity of coffee species. Generally, the heat treatment applied to coffee beans leads to the pyrolysis of organic compounds, forming free radicals and fragments of molecules, which lead to contaminants when they fuse. In coffee, lipids are among the potential precursors, and these range from 10 to 17% of the chemical composition of the bean [103].

Regarding toxic effects, PAHs show potential mutagenicity and carcinogenicity [103,104]. The possibility of genetic damage to humans is mainly associated with high-molecular weight and high-hydrophobicity compounds [101,103,104]. According to the Environmental Protection Agency (US EPA), 16 PAHs are considered of priority due to their frequency of occurrence in food and toxicity (Table 1) [104,105].

According to the European Food Safety Authority (EFSA), four substances (chrysene, benzo(a)anthracene, benzo(a)pyrene, and benzo(b)fluoranthene) are considered markers of the presence of PAHs in food [101]. According to the European Commission, the sum of these four compounds (PAH4) cannot exceed 10 μg/kg for oils and 30 μg/kg for meat and fishing products, with the maximum levels set for benzo(a)pyrene of 2 μg/kg and 5 μg/kg, respectively. There are no maximum limits set for coffee [106].

The analytical techniques most widely used for detecting and quantifying PAHs are high-performance liquid chromatography with fluorescence detector (HPLC-FLD) and gas chromatography coupled to mass spectrometry (GC-MS). In general, the roasting degree does not influence the final concentration of the coffee beverage (infusion), but, in contrast, is an important variable in roasted beans, since the longer the roasting time, the higher the levels of high-molecular weight PAHs. In this sense, choosing light roasting can help in reducing the level of PAHs contamination, especially due to the relatively high volatility of light PAHs [102,103].

### 4.2. Acrylamide

Acrylamide is an acyclic chemical compound of the amide family. Its structure has the NH_2_ radical bound to a carbon atom, which is then bound to an oxygen atom (Figure 5). The acrylamide formation in coffee occurs predominantly due to the reaction between a reducing sugar and the amino acid asparagine, via the Maillard reaction [107]. The cascade of reactions for acrylamide formation can occur from the decarboxylation and deamination of asparagine, forming the contaminant [107]. The unstable Schiff base is formed from the reaction of amino acids with carbonyl compounds and broken up to form acrylamide. This pathway, whereby asparagine transforms into acrylamide, constitutes the first phase of the Maillard reaction [107,108].

In 2002, Tareke et al. reported for the first time the formation of acrylamide in heated foods, and observed that carbohydrate-rich and high-temperature-processed foods had greater potential for the formation of this contaminant [109]. Regarding coffee, studies show that the peak of acrylamide formation occurs in the intermediate stage of the roasting process (160–180 °C), where, predominantly, the Maillard reaction occurs [109,110,111].

The IARC classifies acrylamide as a probable human carcinogen (group 2A) [112]. Its presence in the diet represents a concern, since this substance has carcinogenic, genotoxic, and neurotoxic potential. Coffee is among the foods with the highest acrylamide concentration, with an average value of 522 μg/kg. Roasted ground coffee has lower concentrations than soluble coffee [113,114,115,116]. The European Commission sets benchmark levels for acrylamide content in coffee, which are 400 μg/kg in roasted coffee and 850 μg/kg in soluble coffee [117].

Acrylamide concentrations are higher in light and medium roast coffees. Temperatures above 180 °C favor acrylamide degradation. Thus, lighter roast coffees have higher acrylamide levels, in contrast with dark roasting [108]. Acrylamide formation is significantly different between *Coffea arabica* and *Coffea robusta* species. Acrylamide can be easily transferred to the beverage because it is water-soluble. Granulometry can also interfere in acrylamide extraction, since it helps to increase the contact area between coffee powder and water [118,119,120,121,122].

### 4.3. Furanic Compounds

#### 4.3.1. Furan

Furan (Figure 5) is a heterocyclic organic compound, lipophilic, of low molecular weight (68 g/mol) and transparent liquidity, and with high volatility and a boiling point of 31 °C. This substance has aromatic characteristics due to its structure that accommodates oxygen, which interacts with the unsaturation (double bond) of the aromatic ring, leading to the delocalization of the electron pair [123].

Furan is considered highly dangerous to the organism, associated with genotoxicity, carcinogenicity, and other toxic effects, and is classified as possibly carcinogenic to humans (group 2B) according to the IARC [124,125]. The carcinogenic action of this compound is supposedly explained by metabolic activation by cytochrome P450 in cis-butene-1,2-diol. Due to this worldwide speculation about the depreciable effects on health, researchers have payed special attention to its formation and concentration in food [126,127,128,129].

The thermal degradation of amino acids and carbohydrates, via the Maillard reaction or not, and the thermal oxidation of polyunsaturated fatty acids during roasting can lead to its formation. In coffee, the main precursors of furan are glucose, linolenic acid, and linoleic acid. The furan formation process is related to aldol condensation, cyclization, and the dehydration of intermediates, such as acetaldehyde and glycolaldehyde [126].

Furan concentration in coffees derived from a lighter to a darker roast can vary on average from 2300 to 5375 μg/kg, respectively [126,127,128,129]. There are no regulatory limits to the amount of furan in food. The European Commission recommends (2007/196/EC of 28 March 2007) the monitoring of the contaminant in foodstuffs [128].

Kuballa et al. [130] monitored furan from bean to cup. Even with high volatility and the loss of the contaminant during the beverage extraction process, all infusion methods result in the presence of furan in the final product. Automated coffee machines result in higher concentrations in the beverage when compared with household equipment, due to the greater amount of powder used in the infusion and lower loss by volatility, favoring the concentration of the contaminant. Additionally, the roasting degree (time/temperature), species, and varieties are closely related to the level of furan in the beverage.

#### 4.3.2. Furfuryl Alcohol and 5-Hydroxymethylfurfural

Furfuryl alcohol (FFA) and HMF are process contaminants formed from furan and modified by a hydroxymethyl group. The FFA can account for about 50% of all furans. The HMF is the main volatile compound found in some categories of coffee, such as light roast coffee [131].

The FFA is considered a possible carcinogen to humans (group 2B), according to IARC [132]. The metabolization process of FFA and HMF is associated with sulfotransferases, resulting in 2-sulfoximethylfuran and 5-sulfoximethylfurfural, respectively. These substances are considered mutagenic due to being reactive electrophiles that can interact with DNA molecules or proteins. However, this potential was evidenced in an in vivo study, without evidence in humans [133,134].

Regarding FFA, its formation is not elucidated in a unitary manner in the literature, and some suggested processes include: (i) the retro-aldol cleavage of the isomer of the Amadori product formed via the Maillard reaction; (ii) glucose oxidation; (iii) the cleavage of 1,2-enaminol or 1,2-enediol formed by Maillard reaction or isomerization, respectively. The HMF is associated with 3-deoxyosone, formed via the Maillard reaction and caramelization, in which the aldol reaction between methylglyoxal and glyceraldehyde occurs, or the direct conversion of the fructofuranosyl cation that is formed from fructose/sucrose [135,136].

Studies show that the FFA concentration is higher in lighter roast coffees, and the kinetics of formation of this contaminant are similar to those of other contaminants, such as HMF and acrylamide. The amount of FFA in Arabica coffee is higher than in other coffee species, and this is due to the greater presence of sucrose, which is considered an important precursor and is found in greater quantities in Arabica coffee (7.5%) compared to Robusta coffee (3.5%). Regarding 5-HMF, the literature has already reported that this contaminant increases at higher temperatures, observing a decay in continuous roasting (above 8 min) with temperatures above 220 °C [23,137,138,139,140].

### 4.4. α-Dicarbonyls and Advanced Glycation End Products

The α-dicarbonyls (α-DCs) are compounds of low molecular weight containing two carbonyl groups in the α-carbon. They are intermediates in the caramelization and Maillard reactions, or lipid oxidation. There are 18 α-DCs categories described in foods; however, only glyoxal (GO), methylglyoxal (MGO), and diacetyl (AD) are found in representative concentrations [21,141,142,143,144].

In vitro studies in human cells have shown that GO, MGO, and DA have cytotoxicity due to the inhibition of enzymes important for transcriptional and translational regulation [144]. Additionally, inhaling these compounds during the coffee roasting process can be toxic to humans. The formation of advanced glycation end products (AGEs), which are related to diabetes and kidney disease, occurs from the interaction of α-DCs with the free amino group of proteins. Pentosidine, carboxymethyllysin, and pyrraline are examples of well-characterized and widely studied AGEs [145,146].

The AGEs are formed by non-enzymatic glycation, but they can also originate from glucose self-oxidation, lipid peroxidation, or condensation reactions of the polyol pathway. Reactive α-DCs such as MGO, GO, and 3-deoxyglucosone are preserved species leading to the formation of AGEs. MGO leads to the formation of N-(carboxyethyl)lysine and methylglyoxal-lysine dimer by modifying several proteins. Due to the biological effects that are harmful to humans, the search for natural inhibitors of AGEs formation to treat chronic diseases, such as diabetes mellitus type 2, and decrease their risk is a worldwide priority. There is no recommendation regarding the maximum AGE concentrations in food [145].

Levels of GO, MGO, and DA decrease in darker roasts, and this is probably due to thermal degradation by caramelization and Maillard reactions. α-DCs have also been observed in light-roasted Arabica coffee (200 °C for 10 min), with more evident formation of GO [146].

### 4.5. 4-Methylimidazole

4-methylimidazole (4-MEl) is an organic compound with a heterocyclic structure of simple nitrogen (Figure 5), formed from the reaction of sugars with ammonia. This compound can be formed by the degradation of sugars in alkyl dicarbonyls and alkylketones, which later react with the ammonia released by amino acids and/or proteins via Strecker degradation producing imidazoles, including 4-MEl, during the roasting process [147].

Data from the National Toxicology Program of the United States of America indicate that 4-MEl has been identified as a carcinogenic substance. The study was conducted with mice (males and females) treated with 170 mg of 4-MEI/kg of body weight, and showed that this compound can induce the appearance of pulmonary neoplasia (alveolar/bronchiolar adenoma) and carcinoma [148]. The IARC classified 4-MEl as a possibly carcinogenic substance to humans (group 2B) [149].

The Office of Environmental Health Hazard Assessment (OEHHA) established a tolerable intake of no significant risk level (NSRL) for 4-MEI of 0.05 mg/day body weight [149,150]. Regarding regulation, the European Union recommends a maximum concentration level of 4-MEl of 200 mg/kg for Class III caramels (E150c) and 250 mg/kg for Class IV (E150d) (EU 231/2012). However, no legislation covers the presence of 4-MEl in coffees [150].

The average 4-MEI concentration in Robusta coffee is higher than that in Arabica coffee. Additionally, the concentration of the contaminant increases due to the increase in temperature and roasting time. The 4-MEI levels in decaffeinated coffees compared with soluble coffee are not statistically different, indicating that the decaffeination of raw beans does not affect the amount of its precursors [151].

### 4.6. Chloropropanols and Their Esters

Chloropropanols are a class of chemical contaminants derived from glycerol, and are structurally characterized by three carbon atoms bound to one or two chlorine atoms. Several compounds, including monochloropropanodiols (MCPDs) and dichloropropanols (DCPs), have been identified as contaminants of various foods. Studies show that 3-monochloropropane-1,2-diol (3-MCPD) (Figure 5) is the most abundant chloropropanol, followed by 2-monochloropropane-1,3-diol (2-MCPD), 2,3-dichloropropan-1-ol (2,3-DCP), and 1,3-dichloropropan-2-ol (1,3-DCP), in the proportions of 10%, 1% and 0.1%, respectively, compared with that of 3-MCPD [152,153,154,155].

Chloropropanol esters are a class of molecules that have, in their chloropropanol structure, one or two hydroxyl groups esterified with fatty acids. Analogous to chloropropanols, their esters can be divided into two groups—the MCPD esters (MCPDE), which are the majority and comprise the esters of 3-MCPD (3-MCPDE) and 2-MCPD (2-MCPDE), and the esters of DCPs (DCPE) [152,153,154,155].

The IARC classifies 3-MCDP as a possible carcinogen to humans (group 2B) [154]. An in vivo study showed that 3-MCDP undergoes rapid metabolization and systemic distribution in tissue lipids, such as testicles, brain, liver, and kidneys [155]. According to Lynch et al. [155], the biotransformation of 3-MCDP takes place via two routes, one of which is by the formation of oxalate as a final product by oxidation of 3-MCDP, and the other is by conjugation with glutathione, forming mercapturic acids. The main form of metabolization in mammals is the formation of oxalic acid from the oxidation of 3-MCDP, which begins with the formation of β-chlorolactaldehyde by oxidation, followed by the aldol reaction that converts it to β-chlorolactic acid, then forming oxalic acid. The 3-MCPD esters are completely hydrolyzed by lipase during digestion, leading to the release of free 3-MCPD, which can be absorbed and metabolized as described.

The toxic potential of 3-MCPD has been widely studied. The compound presented nephrotoxicity and adverse effects on reproduction in studies conducted with rats, in addition to carcinogenic, but not genotoxic, potential in high doses (400 ppm). The tolerable daily intake (TDI) for 3-MCPD and its esters was established at 2 μg/kg of body weight by the EFSA Contaminant Panel in 2017, whereas the Joint FAO/WHO Expert Committee on Food Additives (JECFA) determined a value of 4 μg/kg body weight/day for the same parameter [156,157,158,159,160,161].

In food, chloropropanols and their esters are formed from heat treatment above 100 °C, in an environment with low moisture content. The 3-MCPD has already been reported in coffee, and its formation occurs during the roasting process, from intrinsic precursors of the green bean such as lipids and chlorine [160]. The presence of 3-MCPD in coffee is not regulated, but for its occurrence in hydrolyzed vegetable protein and soy sauce, the European Commission establishes maximum levels of 20 μg/kg (for a liquid product containing 40% dry matter) and 50 μg/kg (in dry matter) [162].

The 3-MCPD concentrations in coffee are relatively low, and the highest levels were observed in decaffeinated coffee and products undergoing prolonged roasting. The color is directly linked to 3-MCPD formation, and the darker beans have higher levels of the contaminant. Due to the water dilution effect, 3-MCPD was not detected in coffee beverages [163,164,165,166].

## 5. Mitigation

Mitigation aims to reduce the risk to consumer health from exposure to potentially toxic chemicals, which can be achieved by applying strategies that reduce the formation of these contaminants throughout coffee processing. The literature presents forms of mitigation involving: (i) decreasing the concentration of precursors; (ii) modifying the process conditions; and (iii) eliminating and/or degrading the formed substance post-processing. Table 2 describes some reported mitigation strategies for the previously mentioned compounds.

The strategies from the literature involving reducing the concentration of precursors are basically directed towards acrylamide. Asparagine, considered one of the key precursors in the acrylamide formation, can be removed and/or decreased by the action of the enzyme asparaginase. The enzyme acts on the substrate containing the amino acid asparagine via a nucleophilic attack, thus leading to a dissociation of the amide radical and the release of ammonia, and forming an acyl-enzyme intermediate. In the second phase of the reaction, the intermediate acyl-enzyme is hydrolyzed, forming the L-aspartate molecule. Some authors have observed a decrease in asparagine concentration after the treatment of Arabica and Robusta coffee with the enzyme [167]. The treatment of green coffee beans with asparaginase combined with ultrasound had significant effects on mitigating acrylamide in Robusta coffee without significant sensory interferences [169].

Roasting is the most important step for the formation of contaminants, and its conditions, especially time and temperature, directly affect the concentration of the compounds formed. Decreasing roasting time and temperature can be a form of mitigation for contaminants such as furan and PAHs, but this leads to losses in the sensory aspects of coffee, such as aroma and flavor, since it prevents the formation of volatile substances essential for the final product [113,177]. Hyong et al. [151] observed that as temperature and roasting time increased, MGO, DA, and 4-MEI levels increased significantly. The study by Kwon et al. [146] also used the roast time/temperature ratio, and reported that darker roasting and longer time increased the levels of α-DCs. Regarding 3-MCPD and its esters in soluble coffee, higher concentrations were observed in dark roasting [166,167]. On the other hand, the increase in temperature and roasting time can decrease levels of acrylamide, HMF, and FFA [136,137]. Rattanarat et al. [108] evaluated the PAHs and acrylamide levels in freshly roasted coffee beans using hot air and overheated steam, verifying that the latter can be a method of reducing contaminants due to its oxygen-free heating capacity. Anese et al. [173] investigated the effects of vacuum roasting on acrylamide formation in Arabica beans, and observed a 50% decrease in the concentration of this contaminant for medium roast coffees. Hu et al. [172] reported that the presence of glycine and aspartic acid in raw coffee beans can reduce the formation of acrylamide during coffee roasting.

Regarding mitigation strategies involving the elimination and/or degradation of the formed substance after processing, Wang et al. [180] investigated the action of anthocyanins for furan removal, which resulted in a significant reduction in the contaminant by up to 33%. The use of phenolic compounds (non-phenolic antioxidant, flavonoids, and phenolic acids) in mitigating FFA and HMF is already well established in the literature, with reduction in these contaminants to variable degrees [131]. The use of green technologies such as supercritical CO_2_ extraction demonstrated its maximum efficiency with the removal of 79% of acrylamide in coffee beans [174]. Emerging technologies such as cold plasma and the use of molecular printing polymers and ionic liquids may also present potential for use in the mitigation of some compounds [22,175]. Genovese et al. [176] demonstrated that the furan levels decreased due to grinding during the degassing phase, possibly due to this process increasing the surface area and opening the cellular structures, facilitating the release and thus the loss of furan. However, the finer grinding of coffee beans as a mitigation measure can be problematic for the preparation of beverages that require thicker grinding. For espresso coffee, using smaller particles led to significantly higher α-DCs and 4-MEI concentrations, whereas for cold brew, the highest α-DCs and 4-MEI concentrations were verified with larger coffee bean particles [151]. The concentrations of some contaminants can also be decreased depending on the brewing method, which may represent a viable alternative at the consumer level [124,177].

As can be seen, the studies described in the literature demonstrate different ways to reduce the occurrence of thermal contaminants in coffee induced by roasting. However, little information is presented about the advantages and disadvantages of these strategies in relation to aspects such as costs, potential for application on an industrial scale and impacts on sensory properties. Optimizing roasting conditions (time/temperature), grinding and brewing procedures could be more cost-effective strategies, since changes in equipment and the use of emerging technologies require financial investments. However, it should be noted that the use of higher roasting temperatures to reduce the concentration of some contaminants, such as acrylamide, may result in an increased degradation of polyphenols and/or deterioration of the sensory characteristics and antioxidant activity [171]. Most of the strategies presented were evaluated only on a laboratory scale, with the use of asparaginase being one of the most promising and effective methods at the industrial scale, since it has already been used for other food matrices (e.g., biscuits) [182]. Regarding the impacts on sensory characteristics, few authors conducted related studies [169,173,175,180], but there are still many biases related to the mitigation methods due to the lack of experiments involving the determination of substances that are relevant to the organoleptic properties.

## 6. Conclusions

Coffee is a product commercialized and much appreciated worldwide. Due to the presence of natural chemical constituents and the need for high temperatures applied in the roasting process, several toxic compounds can be formed in coffee, with carcinogenic and genotoxic potential, among others, representing a public health concern. Therefore, knowing the precursors of these contaminants and their mechanisms of formation is important to developing efficient mitigation strategies. However, many challenges must still be overcome to make coffee a safer and higher-quality product regarding the presence of thermal contaminants induced by roasting. First, it is necessary to find means for simultaneously reducing these contaminants, since the same strategy can disfavor the formation of a group of compounds and at the same time favor the formation of others. Additionally, most studies have been conducted on a laboratory scale, and need to be tested on an industrial scale. Finally, the impacts of implementing mitigation strategies on the sensory aspects of the product are not well known. It is important that the levels of these contaminants be continuously monitored in order to allow the assessment of the real risks that they pose to human health.

## Figures and Tables

**Table 1:** Polycyclic aromatic hydrocarbons (PAHs) considered as a priority by the US Environmental Protection Agency and their IARC classification.

Substance	Genotoxicity	IARC Classification	Aromatic Rings	Structure
Naphthalene	Positive	2B	2	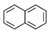
Acenaphthene	Questionable	Not evaluated	3	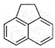
Acenaphthylene	Questionable	Not evaluated	3	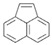
Anthracene	Negative	3	3	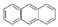
Phenanthrene	Questionable	3	3	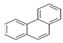
Fluoranthene	Positive	3	4	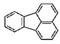
Fluorene	Negative	3	3	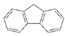
Pyrene	Questionable	3	4	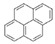
Benzo(a)anthracene	Positive	2B	4	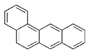
Crysene	Positive	2B	4	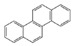
Benzo(b)fluoranthene	Positive	2B	5	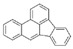
Benzo(k)fluoranthene	Positive	2B	5	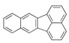
Dibenzo(a,h)anthracene	Positive	2A	5	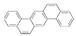
Benzo(a)pyrene	Positive	1	5	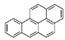
Indene(1,2,3-cd)pyrene	Positive	2B	6	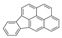
Benzo(g,h,i)perylene	Positive	3	6	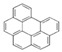

Source: Park and Penning (2009) [105]; reproduced with permission from John Wiley & Sons, title Process-Induced Food Toxicants: Occurrence, Formation, Mitigation, and Health Risks.

**Table 2:** Mitigation strategies for thermal contaminants in coffee induced by roasting.

Technology for Reduction	Contaminant	Reduction Level	Reference	Comments	Limitations
Asparaginase	Acrylamide	60% reduction in asparagine in *C. arabica* and 35% in *C. robusta*.	[167]	This study aimed to test the efficacy of asparaginase application and evaluate the reduction in asparagine in coffee beans. The results showed the effectiveness of the enzyme in reducing free asparagine in green coffee beans of both species (*C. arabica* and *C. robusta*).	Steam pretreatment was effective for the two species but required different times (30 min for *C. arabica* and 45 min for *C. canephora*). This can be attributed to the different chemical compositions found in the two species.
Asparaginase	Acrylamide	59% reduction in acrylamide in Arabica coffee compared to the control sample.	[168]	The predicted reduction was obtained with a hydrolysis time of 2 h, water content of 90% and asparaginase load of 5000 ASNU (amount of asparaginase that produces one micromole of ammonia per minute under standard conditions (pH 7.0; 37 °C))/kg. The applied method did not influence the content of caffeine, chlorogenic acid or caffeic acid.	Impacts on the sensory profile were not evaluated.
Asparaginase treatment combined with ultrasound	Acrylamide	Reductions of 42% and 14% in acrylamide in the final product in relation to the reference sample (7 min roasting at 220 °C) and control sample (without asparaginase treatment), respectively.	[169]	Pretreatment conditions of green coffee: enzyme concentration of 3 IU/mL, immersion time of 30 min, temperature of 37 °C, pH 7.3 and ultrasound with power of 37 kHz.	At enzyme concentrations higher than 3 IU/mL, the decrease in acrylamide concentration in the final product was not observed. This may be due to the fact that the penetration of the enzyme into the beans is not favored at high concentrations, leading to a decrease in the asparaginase efficiency.
Asparaginase	Acrylamide	Not reported.	[170]	Arabica green coffee beans were treated with L-asparaginase at various dosages (0–4000 ASNU/kg green bean) and times (30–120 min). The optimal conditions were determined as 2126.4 ASNU/kg of green bean and 82.73 min. A moderate positive correlation (r = 0.669, *p* < 0.01) was found between the concentration of asparagine in green coffee beans and the acrylamide content of coffees produced from these beans.	The optimum treatment time (82.75 min) was higher than the treatment time recommended for the commercial enzyme (45–60 min).
Roasting conditions	Acrylamide	Not reported.	[171]	In the roasting of Robusta coffee, temperatures ranging from 190 to 216 °C and dry or moist air at a velocity of 0.5 or 1 m/s were used. The optimum roasting parameters were: temperature 203 °C, dry air and low speed of the roasting air. Under these conditions, roasted beans were characterized by relatively low levels of acrylamide with moderate degradation of polyphenols and deterioration of antioxidant properties, while having a pleasant, full flavor.	The reduction in acrylamide formation caused by the increase in humidity of roasting air was observed only at the highest roasting temperature. Modifying the roasting conditions to achieve a drop in acrylamide concentration resulted in the increased degradation of polyphenols and/or deterioration of antioxidant activity.
Roasting conditions	Acrylamide	Not reported.	[172]	Robusta coffee beans were roasted in a hot air roaster, in which the temperature was increased from room temperature to 220 °C. The maximum acrylamide level of 974 ± 29 μg/kg was achieved at 180 °C. Thereafter, acrylamide continuously decreased down to 109 ± 18 μg/kg. In a model system, the addition of glycine and aspartic acid exerted an inhibitory effect on acrylamide production (up to 27% and 25%, respectively).	The effects of amino acids on acrylamide formation were evaluated via an asparagine–FGS (fructose/glucose/sucrose) model system only.
Roasting conditions	PAHs	Eight-fold decrease in the 19 PAHs content in Arabica coffee beans from Indonesia (42.32 to 5.57 μg/kg), Kenya (from 57.65 to 8.17 μg/kg) and Tanzania (from 59.0 to 8.05 μg/kg). Robusta coffee, on the other hand, had a decrease of about six times for the 19 PAHs from Cameroon (from 58.36 to 9.29 μg/kg) and Indonesia (from 48.52 to 8.57 μg/kg).	[20]	The temperature and time variables used were 125–135 °C for 25–26 min. The contamination level for 19 PAHs varied from 4.29 to 16.17 µg/kg in roasted coffee beans and from 8.66 to 76.63 µg/kg in green coffee beans. The contamination level in roasted coffee beans was significantly lower than that in green beans. The roasting process itself significantly reduced the PAHs content in the final product and the reason for this phenomenon was relatively high volatility of light PAHs.	The geographical origin of green coffee beans, the local environmental pollution and the method of post-harvest coffee drying cause significant differentiation in the PAHs’ initial contamination level.
Superheated steam (SHS)	PAHs and acrylamide	Decrease in acrylamide content in medium (~16%) and dark roast (~25%) coffee beans; decreased PAHs levels in roasted coffee beans (62–69%).	[108]	SHS can help to reduce the contaminants due to its oxygen-free heating capacity. The same roasting parameters were used in the beans, with temperatures of 210, 230 and 250 °C, until the values of color reached 30, 25 and 20, which indicate the light, medium and dark levels of roasting, respectively.	SHS roasting at 250 °C decreased the acrylamide contents in medium- and dark-roasted coffee beans, but in the case of the light-roasted beans, SHS roasting seemed to induce more extensive acrylamide formation than hot air roasting. This is probably because SHS roasting led to significantly higher contents of acrylamide precursors, especially glucose, which was generated by the thermal hydrolysis of carbohydrates during the earlier stage of roasting. SHS roasting at 250 °C can be used to lower PAH contents only in dark-roasted coffee beans. Further studies involving industrial application of the proposed mitigation strategy as well as its energy consumption and cost should be conducted.
Vacuum roasting	Acrylamide	~50% decrease.	[173]	*Coffea arabica* beans were roasted in an oven at 200 °C for increasing lengths of time under vacuum (i.e., 0.15 kPa). Data were compared with those obtained from coffee roasted at atmospheric pressure (i.e., conventional roasting), as well as at atmospheric pressure for 10 min followed by vacuum treatment (0.15 kPa; i.e., conventional vacuum roasting).	No significant differences in acrylamide concentration among the coffee beans roasted according to the different processes were found at thermal effect (*F*) values higher than 15 min. The vacuum dark-roasted coffee presented a lower odor intensity compared with the reference sample (conventionally roasted). Further research should be conducted at pilot and industrial scale to find optimum process conditions.
Supercritical CO_2_ extraction	Acrylamide	79% decrease.	[174]	The acrylamide removal efficiency ranged from 8% to 45% when the extraction duration was less than 525 min, which corresponds to a supercritical solvent consumption of approximately 19 g/g of coffee beans. When the extraction time was increased to 1305 min, a maximum extraction efficiency of 79% was found, which corresponds to a supercritical solvent consumption of 47 g/g of coffee beans. Temperature was the variable that most affected the measured extraction process, as the extraction efficiency was highly increased above 60–80 °C. The addition of ethanol (up to 9.5% w/w) resulted in a significant increase in extraction performance due to the change in polarity of the supercritical solvent mixture. The best working conditions found were 100 °C, 200 bar and 9.5% w/w of ethanol.	The authors did not evaluate the optimization of the acrylamide extraction process with the organoleptic investigation. Exploratory degustation tests pointed out that the supercritical removal of acrylamide must be followed by a cleaning step with pure supercritical CO_2_ in order to remove the ethanol residues.
Corona discharge plasma jet (CDPJ)	Benzo[a]pyrene and acrylamide	Decreases of 53.6% and 32% for benzo[a]pyrene and acrylamide, respectively.	[175]	CDPJ was generated under atmospheric pressure conditions using a 1.5 A current (input) at 58 kHz operating frequency. For each treatment, roasted coffee beans taken in a Petri plate were exposed to CDPJ. The plasma treatment times were 0, 15, 30, 45, and 60 min. There was no change in components such as total acids, caffeine and trigonelline.	After CDPJ treatment, the concentration of total phenolic content and Agtron color values were altered significantly. The CDPJ treatment of beans altered aroma and aftertaste characteristics of the corresponding coffee brews.
Use of phenolic acids, flavonoids, non-phenolic antioxidants, and non-antioxidant agents	Furfuryl alcohol (FFA) and 5-hydroxymethyl- furfural (HMF)	Variable.	[131]	Ascorbic acid showed good mitigating effects (in the range of 48.3–80.7%). No mitigating effect of β-carotene on FFA formation was observed. Sodium sulfite drastically reduced HMF by 87.7%, while it had no effect on FFA. Taurine exhibited a notable effect only on FFA by a 39.6% mitigation.	The composition of model systems during mitigation experiments should be carefully chosen, since components that are presumed to be non-precursors can adversely interfere with the work of mitigation agents in the model systems.
Particle size and species	Furan	11.4% decrease.	[176]	In the Arabica coffee, the furan level in espresso coffee increased with increasing particle size. Particularly, from circumference (C) < 200 μm to C > 425 μm fractions, furan increased from 68.27 to 91.48 ng/mL. In Robusta coffee, the highest concentration of furan occurred in beverages prepared using C 300–425 μm, showing values of 116.39 ng/mL and 845.14 ng/mL, respectively.	*Robusta* coffee reached a concentration of furan around 35% higher than *Arabica* coffee for both intermediate size classes C200–300 μm and C300–425 μm.
Roasting conditions and particle size	4- methylimidazole (4-MEI) and α-dicarbonyls (α-DC)	Not reported.	[151]	With increasing temperature and roasting time, levels of methylglyoxal (MGO), diacetyl (DA) and 4-MEI increased significantly (*p* < 0.05). In the espresso beverage, the use of smaller coffee bean particles led to significantly higher concentrations of α-DC and 4-MEI (*p* < 0.05). In the cold brew method, the highest concentrations of α-DC and 4-MEI were found with the largest coffee bean particles (*p* < 0.05). Among the α-DCs, MGO was present in the greater quantity, followed by glyoxal (GO) and DA.	The effect of the particle size as a mitigation strategy depends on the type of beverage. Further sensory evaluation studies are needed to assess the taste experienced by consumers.
Brewing method	PAHs	Not reported.	[177]	PAHs were evaluated in ground roasted coffees in three roasting degrees obtained from *Coffea arabica* and *Coffea canephora* and their respective coffee brews prepared by two brewing procedures (filtered and boiled). Filtered coffee presented average summed PAHs contents higher than boiled coffee.	Coffee brews prepared with *C. arabica* ground roasted beans presented mean summed PAHs levels higher than the ones prepared with *C. canephora*, independently of the brewing procedure used. A high variability of the results within the same cultivar and roasting degree, submitted to the same brewing procedure, was verified.
Brewing method	Furan	Not reported.	[124]	Furan was evaluated in filtered nonboiled coffee (prepared by letting water (92−96 °C) drip onto roasted ground coffee held in a paper filter) and filtered boiled coffee (prepared by mixing water (25 °C) with roasted ground coffee, heating the mixture until boiling, and filtering it in a paper filter). Most of the beverages made by filtering nonboiled coffee showed significantly higher furan levels than beverages made by filtering boiled coffee. This could be due to a higher furan volatilization when the ground coffee is boiled with the water before filtration, resulting in a higher % of furan reduction in these beverages.	Factors other than the brewing procedure may influence the furan content in the beverages such as the food composition, especially the lipid content.
Acrylamidase	Acrylamide	~97% decrease	[178]	Immobilized acrylamidase could degrade acrylamide from roasted instant coffee solution within 60 min at initial concentrations of 100–500 mg/L in packed column operations. Acrylamidase can hydrolyze preformed acrylamide to acrylic acid and ammonia.	The reusability of immobilized enzyme showed that about 70% of its initial activity was retained after four consecutive cycles of acrylamide degradation. The activity decreased on further reuse and reached 30% after seven cycles. The reduction in enzyme activity may be due to the shear forces exerted on the beads during column operations, or due to the accumulation of the coffee constituents at the active site of the enzyme.
Fermentation	Acrylamide and HMF	70% for acrylamide and up to 99.2% for HMF	[179]	Instant coffee (20%, w/v) was mixed with sucrose (0–10, w/v) and baker’s yeast (*Saccharomyces cerevisiae*, 1–2%, w/v) in a tightly closed glass vessel. The mixture was fermented at 30 °C for 48 h. HMF and acrylamide contents were reduced exponentially at varying rates, depending upon fermentation medium and time. After 24 h, the HMF concentration was decreased by 61.2%, 75.7%, 93.6% and 99.2% in the fermentation media containing no, 1%, 5%, and 10% sucrose, respectively. After 48 h, acrylamide concentration was decreased by about 70%.	Increasing the yeast concentration to 2% improved slightly the mitigation of acrylamide during the fermentation. The metabolic degradation product of acrylamide is unknown for *S*. *cerevisiae* and needs further investigation.
Red-fleshed apple anthocyanin extract (RAAE)	Furan	Up to 33%.	[180]	The addition of RAAE significantly reduced the content of furan in ground coffee. Anthocyanins are effective antioxidants, and they effectively burst free radicals and terminate the chain reaction of oxidative damage.	The reduction of furan content in coffee by RAAE was closely related to its antioxidant capacity.
Organic selenium	Acrylamide	73% decrease.	[181]	Organic selenium as antioxidant additive supplemented via pretreatment of green coffee beans was effective in reducing acrylamide formation by 73%. The increase in antioxidation capacity from selenium fortification and removal of water-soluble precursors of the Maillard reaction may explain the acrylamide reduction mechanism of Se-coffee.	For higher selenium concentrations, ~30% selenium loss was observed after roasting the Arabica and Robusta Se-coffee beans. Robusta coffee showed a significantly lower final selenium content after roasting. Besides costing, sensory properties of the Se-coffee remain the most important consumer parameter that warrants study.

## Data Availability

Not applicable.
